# Ultrasound pretreatment enhances drying efficiency and phenolic retention in raspberries during heat pump drying by modulating cell wall structure and water status

**DOI:** 10.1016/j.fochx.2026.104213

**Published:** 2026-07-14

**Authors:** Yi Li, Simeng Wen, Xilei Sun, Junjie Chen, qiaoyu Yang, Jiahe Dai, Lijing Liu, Hong Li, Kunhua Wang

**Affiliations:** a College of Food Science and Technology, Yunnan Agricultural University, Fengyuan Road 452, Kunming 650201, China; b Yunnan College of Coffee Modern Industry, Yunnan Agricultural University, Fengyuan Road 452, Kunming 650201, China

**Keywords:** Raspberry, Ultrasound pretreatment, Heat pump drying, Cell wall structure, Water mobility, Phenolic retention

## Abstract

Prolonged drying time and substantial loss of bioactive compounds remain major challenges in raspberry drying. This study investigated the effects of ultrasound pretreatment (45 kHz, 500 W, 45 °C, 5–20 min) on cell wall structure and physicochemical properties, water status, drying behavior, and phenolic content during heat pump drying. Ultrasound disrupted raspberry tissue structure, increased accessible surface area, reshaped pore size distribution, promoted pectin solubilization and depolymerization, reduced pectin molecular weight, and enhanced water mobility. These structural changes likely reduced internal resistance to water transport, which in turn shortened the drying time from 8.87 h in the control group to 5.53 h after 15 min treatment, representing a 37.7% reduction. Fifteen phenolics were identified, and US-15 showed the best preservation of anthocyanins, whose contents were 8.46%–25.52% higher than those in control group. Overall, ultrasound pretreatment improved drying efficiency and phenolic retention by remodeling cell wall architecture and promoting water redistribution.

## Highlights

• Ultrasound promoted the solubilization and depolymerization of pectin.
• Ultrasound disrupted tissue integrity and increased cell-wall matrix porosity.
• Ultrasound induced the side chains breakage of pectin.
• Increased cell wall porosity and greater water freedom facilitated drying.
• Ultrasound significantly increased anthocyanin content in dried product.

## 1 Introduction

Raspberries (*Rubus idaeus* L.) are widely valued for their distinctive flavor and high nutritional quality, particularly their abundance of phenolics such as anthocyanins, ellagic acid, and ellagitannins (Lopez-Corona et al., 2022). These phytochemicals are closely associated with the antioxidant and health-promoting properties of raspberry products. However, fresh raspberries are highly perishable because of their soft texture, delicate tissue structure, and high moisture content, which make them particularly susceptible to mechanical damage, microbial spoilage, and to rapid postharvest deterioration (Piccolo et al., 2020). Drying is therefore an important preservation strategy for extending shelf life and improving the stability, transportability, and marketability of raspberries (Gao et al., 2023). Nevertheless, conventional drying processes are often limited by low drying efficiency and severe quality deterioration, including prolonged drying times and losses of thermolabile phenolics (Belwal et al., 2022).

Heat pump drying has emerged as a promising alternative for fruit dehydration because of its high energy efficiency and relatively mild operating temperature (Zhu et al., 2025). Compared with conventional hot-air drying, heat pump drying can better preserve heat-sensitive compounds and improve product quality (Loemba et al., 2023). However, for raspberries, the high initial moisture content and compact internal tissue still impose considerable resistance to internal water diffusion, resulting in a prolonged drying period and significant degradation of thermally sensitive bioactive compounds (Pateiro et al., 2022). Therefore, improving internal mass transfer remains a key challenge in raspberry heat pump drying, and suitable pretreatment strategies are needed to accelerate moisture migration while minimizing quality loss (Miraei Ashtiani et al., 2023). Ultrasound pretreatment has attracted increasing attention as a green and non-thermal intensification technology for fruit and vegetable drying. In an aqueous medium, ultrasound generates cavitation, microstreaming, mechanical vibration, and the sponge effect, which can disrupt plant tissues, weaken structural barriers, and create microchannels for water transport (Zhou et al., 2024). Previous studies have shown that ultrasound pretreatment can effectively shorten drying time and improve certain quality attributes of plant materials, such as mulberries (Wang, He, Wang, et al., 2024) and goldenberries (Miraei Ashtiani et al., 2022). However, in most cases, the positive effect of ultrasound has been described mainly at the phenomenological level, whereas the structural basis of drying enhancement remains insufficiently understood. In particular, for raspberry heat pump drying, the relationship between ultrasound-induced cell wall remodeling, water redistribution, and final phenolic retention has not yet been systematically clarified.

The plant cell wall is a major structural determinant governing both tissue integrity and moisture migration. It is composed mainly of cellulose (CL), hemicellulose (HC), and pectin, among which pectin plays a dominant role in cell-to-cell adhesion and middle lamella integrity (Xian et al., 2024). Alterations in pectin solubility, molecular size, branching characteristics, and degree of esterification (DE) can substantially modify the porosity and permeability of the cell wall matrix, thereby influencing water mobility during drying (Niu et al., 2024; Sun et al., 2026). Ultrasound may accelerate these structural modifications by promoting pectin solubilization and depolymerization, loosening the cell wall network, and enlarging internal transport pathways (Wang, He, Wang, et al., 2024). Such changes are expected to alter the state and spatial distribution of water in raspberry tissues, facilitating the conversion of more immobilized water into more mobile water and ultimately enhancing drying kinetics. However, direct evidence linking these structural changes to drying performance in raspberries remains limited. Previous ultrasound-assisted drying studies have established that acoustic cavitation and the sponge effect can promote moisture removal by disrupting plant tissues and forming microchannels (Miraei Ashtiani et al., 2022; Wang, He, Wang, et al., 2024; Zhou et al., 2024). Recent studies have further linked ultrasound-induced cell-wall loosening and enhanced water mobility to improved drying performance in some fruits (Niu et al., 2024; Sun et al., 2026; Wang, He, Wang, et al., 2024). However, these studies have generally focused on drying kinetics, effective moisture diffusion, or bulk quality indicators, whereas the sequential relationship among pectin fraction transformation, pore-structure redistribution, water-state conversion, and the fate of individual phenolics remains insufficiently resolved within a single experimental framework. This gap is particularly relevant to raspberries because their soft drupelet structure, high anthocyanin content, and ellagitannin-rich matrix make their drying quality highly sensitive to both thermal exposure and cell-wall disruption.

In addition to mass transfer, cell wall modification may also be closely related to phenolic retention during drying (Niu et al., 2025). Phenolics can associate with cell wall polysaccharides through non-covalent interactions, and disruption of the cell wall matrix may affect both the release and stability of these compounds (Liu et al., 2020). Meanwhile, a faster drying process may reduce thermal exposure and help preserve thermolabile anthocyanins. Therefore, ultrasound pretreatment may influence phenolic retention through two interconnected pathways: by shortening drying time and by altering the physicochemical environment of phenolics within the tissue matrix. Clarifying these relationships may help explain how drying conditions shape product quality and facilitate the development of pretreatment strategies that simultaneously improve drying efficiency and nutritional quality.

Accordingly, the present study aimed to systematically investigate how ultrasound pretreatment modulates the heat pump drying behavior and phenolic retention of raspberries from the perspective of cell wall structure and water status. This work provides mechanistic insight into how ultrasound-assisted pretreatment enhances raspberry drying and offers a theoretical basis for improving the efficiency and quality of dried berry products.

## 2 Materials and methods

### 2.1 Plant material

Fresh raspberries (*Rubus idaeus* L.) were obtained from the Qingsongling Raspberry Planting Base in Kunming, Yunnan Province, China. The fruits were harvested at commercial ripeness on 15 July 2025, with a total soluble solids (TSS) content of approximately 11.5°Brix and an initial moisture content of 85.75% (TSS and initial moisture content were determined as described in the Supplementary Materials). After harvest, the raspberries were transported to the laboratory within 2 h under insulated conditions and processed on the same day. The fruits were not frozen before the experiments and were temporarily stored at 4 °C before treatment to minimize postharvest deterioration. Prior to experimentation, damaged, defective, or overripe fruits were removed, and the remaining samples were washed, air-dried, gently pooled, and randomly allocated to different treatment groups. No significant differences (*p* > 0.05) in TSS and initial moisture content were observed among the treatment groups.

### 2.2 Experimental design

A total of 40 kg of raspberries were randomly divided into five groups, and all experiments were conducted in triplicate. Ultrasound pretreatment was performed using a 500 W ultrasonic bath (KQ-500E, Kun Shan Ultrasonic Instruments Co., Ltd., Suzhou, China). The raspberries were immersed in distilled water at a sample-to-water ratio of 1:4 (*w*/*v*) and treated at 45 kHz and 45 °C for 5, 10, 15, or 20 min. Untreated raspberries served as the control group. According to the ultrasound duration, the samples were designated as CON (untreated control), US-5, US-10, US-15, and US-20. Preliminary experiments indicated that water immersion at 45 °C did not significantly affect the drying time or phenolic content of raspberries. The initial moisture content of raspberries did not differ significantly (p > 0.05) between the ultrasonic treatment groups and the non-ultrasonic control group.

### 2.3 Texture measurement

Texture profile analysis (TPA) was performed using a texture analyzer (TA.XT Plus, Stable Micro Systems Ltd., Godalming, UK) according to the method of Wang et al. (2022), with slight modifications. Individual raspberries were subjected to a two-cycle compression test using a 36-mm aluminum cylindrical probe with a radiused edge (P/36R). The pre-test, test, and post-test speeds were set at 1.0, 1.0, and 5.0 mm/s, respectively. Each fruit was compressed to 40% of its original height. Hardness (N) was defined as the maximum force during the first compression cycle. Gumminess (N) was calculated as hardness × cohesiveness, representing the force required to disintegrate a semi-solid sample to a swallowable state, while chewiness (N) was calculated as gumminess × springiness, representing the energy required to chew a sample before swallowing. Ten raspberries were randomly selected from each treatment group for analysis.

### 2.4 SEM and TEM observations

The ultrastructure of raspberry tissues was examined using scanning electron microscopy (SEM; SU3500, Hitachi High-Tech Corporation, Tokyo, Japan) and transmission electron microscopy (TEM; Tecnai G2 F20, FEI Company, Hillsboro, OR, USA). For SEM analysis, raspberry tissues were cut into small blocks of approximately 5 × 5 × 3 mm^3^ and fixed in 2.5% glutaraldehyde prepared in 0.1 M phosphate buffer (pH 7.2) at 4 °C overnight. After fixation, the samples were rinsed three times with phosphate buffer and dehydrated through a graded ethanol series of 30%, 50%, 70%, 80%, 90%, 95%, and 100%. The dehydrated samples were freeze-dried, mounted on aluminum stubs with conductive adhesive tape, sputter-coated with gold, and observed by SEM (Wang, Li, Xue, et al., 2024).

For TEM analysis, raspberry tissue blocks were trimmed into approximately 1 mm^3^ pieces and fixed in 2.5% glutaraldehyde in 0.1 M phosphate buffer (pH 7.2) at 4 °C overnight. The samples were rinsed with the same buffer, post-fixed with 1% osmium tetroxide for 1–2 h, dehydrated through a graded ethanol series, and embedded in epoxy resin. Ultrathin sections of approximately 70 nm were prepared, stained with uranyl acetate and lead citrate, and observed by TEM (Wang, Li, Xue, et al., 2024).

### 2.5 Determination of cell-wall polysaccharide fractions

Alcohol-insoluble residue (AIR) was prepared from raspberry pulp based on Zhang et al. (2025). Briefly, homogenized pulp was treated with 95% ethanol and boiled to inactivate enzymes, then washed with ethanol and acetone, and dried at 40 °C. The AIR was sequentially extracted to obtain different polysaccharide fractions: water extraction yielded water-soluble pectin (WSP), followed by CDTA for chelate-soluble pectin (CSP), Na₂CO₃ with NaBH₄ for sodium carbonate-soluble pectin (NSP), and NaOH with NaBH₄ for HC, while the final residue was regarded as CL. All extracts were filtered, dialyzed, freeze-dried, and experiments were performed in triplicate.

Pectin content was quantified as galacturonic acid (GalA) using the meta-hydroxydiphenyl method at 530 nm. The contents of HC and CL were determined by the anthrone colorimetric method at 620 nm, as described by Sun et al. (2026). All measurements were performed in triplicate.

### 2.6 Pore size distribution and specific surface area of cell walls

The pore size distribution and specific surface area (SSA) of raspberry cell walls were determined using an Autosorb-iQ automatic surface area and pore size analyzer (Autosorb-iQ, Quantachrome Instruments, Boynton Beach, FL, USA), following the method of Jiang et al. (2023). Briefly, 0.25 g of freeze-dried raspberry powder was used for nitrogen adsorption analysis at liquid nitrogen temperature. The pore size distribution was calculated using the Barrett-Joyner-Halenda method, whereas the SSA was calculated using the Brunauer-Emmett-Teller method.

### 2.7 Molecular weight (mw) of pectin fractions

The Mw of the three pectin fractions was determined using a gel permeation chromatography system (Agilent 1260 Infinity II, Agilent Technologies Inc., Santa Clara, CA, USA) equipped with Ultrahydrogel TM120, TM250, and TM500 water-soluble gel columns (7.8 mm × 300 mm; Waters Corporation, Milford, MA, USA). The chromatographic conditions followed the method described by Xing et al. (2023).

### 2.8 DE of pectin fractions

The DE was determined by titration according to Liang et al. (2022). The sample was first titrated with 0.1 M sodium hydroxide, followed by the addition of 20 mL of 0.5 M sodium hydroxide and incubation for 15 min. Subsequently, 20 mL of 0.5 M hydrochloric acid was added, and the sample was shaken until the pink color disappeared. The sample was then titrated again with 0.1 M sodium hydroxide to a pale pink endpoint. A blank control was prepared under the same conditions. The DE was calculated based on the volume of sodium hydroxide consumed. All measurements were performed in triplicate.

### 2.9 Neutral sugar composition of pectin fractions

The neutral sugar composition of the pectin fractions was determined according to Li et al. (2025). Different pectin fractions were hydrolyzed with 2 mol/L trifluoroacetic acid at 121 °C for 2 h. The hydrolysates were then dried under nitrogen, redissolved in distilled water, and transferred to chromatography vials. Neutral sugars were analyzed by ion-exchange chromatography using an ICS5000+ system (Dionex ICS-5000+, Thermo Fisher Scientific Inc., Waltham, MA, USA) equipped with a Dionex CarboPac PA20 column (150 × 3.0 mm, 10 μm).

The calculation methods for R1, R2, and R3 were as follows (Xing et al., 2023). Ratio 1 (R1) = (GalA-Rha)/(GalA + Rha + Ara + Gal + Xyl) × 100; Ratio 2 (R2) = (2Rha + Ara + Gal)/(GalA + Rha + Ara + Gal + Xyl) × 100; Ratio 3 (R3) = (Gal +Ara)/Rha.

### 2.10 Low field nuclear magnetic resonance (LF-NMR) and magnetic resonance imaging (MRI) analysis

The water status of raspberries was analyzed using a LF-NMR analyzer coupled with MRI (PQ21-040 V, Suzhou Niumag Analytical Instrument Co., Ltd., Suzhou, China) with the CPMG sequence, according to the method of Wang, He, Wang, et al. (2024). The operating parameters were as follows: proton resonance frequency, 20 MHz; spectral width, 100 kHz; repetition time, 2000 ms; digital gain, 3; and pulse widths P1 and P2 of 5.10 and 9.04 ms, respectively. Each measurement was performed in triplicate. Detailed information on the inversion procedure for T₂ relaxation analysis and the identification of the three water populations (T₂₁, T₂₂, and T₂₃) has been provided in the Supplementary Materials.

### 2.11 Heat pump drying

**Fig. 1:**
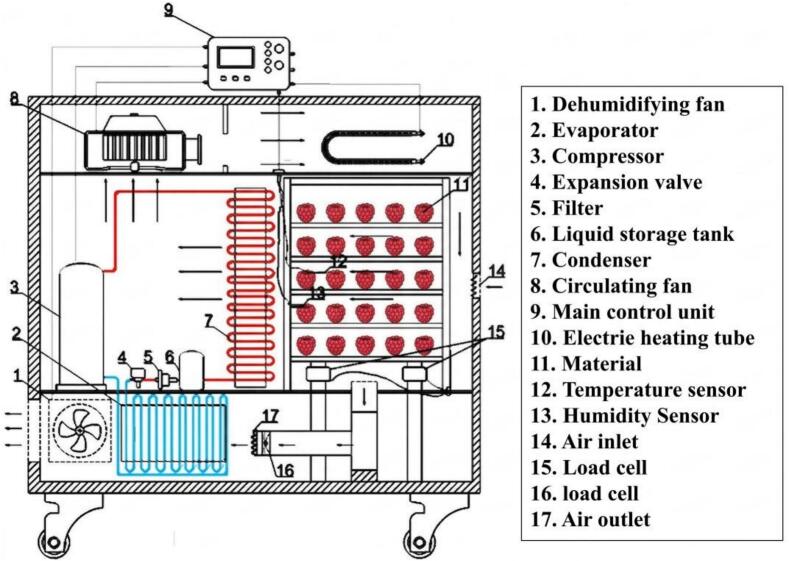
Schematic diagram of heat pump drying equipment.

Control and ultrasound-pretreated samples were subsequently dried. For each run, approximately 1 kg of fruit from each group was evenly spread in a single layer on a wire mesh tray. The heat pump drying system (AHRD023-DX, Henan Bogui Machinery Co., Ltd., Henan, China), installed at Yunnan Agricultural University, Kunming, China, consisted mainly of a drying chamber, a heating unit (condenser and heating tube), a dehumidification unit (evaporator and dehumidifying fan), an air circulation unit, and a control unit (Fig. 1). Additional technical parameters of the heat pump drying system are provided in the Supplementary Materials. Based on preliminary experiments, the drying conditions were set at 60 °C, an air velocity of 2 m/s, and a relative humidity of 15%. Changes in sample weight during drying were recorded using the built-in weight sensor integrated into the system. Drying was terminated when the sample moisture content fell below 15% (wet basis), which was selected as the target endpoint to ensure comparable dehydration levels among treatments and to balance storage stability with quality preservation in dried raspberries (Gao et al., 2023). Each treatment was performed in triplicate.

### 2.12 Drying behavior

The moisture ratio (*MR*) of the samples during the drying process was determined using Eq. (1) (Zhong et al., 2025):

(1) $$\mathrm{MR}=\frac{M_{t}-M_{e}}{M_{0}-M_{e}}$$

where *M*_t_, *M*_0_, and *M*_e_ are moisture content at time *t*, time 0, and equilibrium, respectively, g/g (dry basis).

The drying rate (*DR*) was calculated according to Eq. (2) (Zhong et al., 2025):

(2) $$\mathrm{DR}=\frac{M_{t}-M_{t+\Delta t}}{\Delta t}$$

*M*_t_, and *M*_t+Δt_ are the moisture contents at times t and t + Δt, respectively, and Δt is the time interval between two samplings (h).

### 2.13 Determination of phenolics content

Phenolics were extracted according to Yu et al. (2021), with slight modifications. Freeze-dried raspberry powder (0.5 g) was ultrasonically extracted three times with 15 mL of acidified methanol (1% HCl; 24 kHz, 450 W, 30 min). After centrifugation at 4000 rpm for 15 min at 4 °C, the combined supernatants were evaporated at 45 °C, redissolved in 10 mL of methanol, filtered through a 0.45 μm membrane, and stored at −40 °C as the free phenolic fraction.

The phenolic extracts were analyzed using high-performance liquid chromatography-electrospray ionization-quadrupole time-of-flight mass spectrometry (HPLC-ESI-qTOF-MS). HPLC-ESI-qTOF-MS system consisted of an Agilent 1290 Infinity II LC system coupled to an Agilent 6545 Q-TOF LC/MS system (Agilent Technologies Inc., Santa Clara, CA, USA). Before analysis, the samples were filtered through a 0.22 μm membrane. Separation was achieved on a ZORBAX SB C18 column (250 mm × 4.6 mm, 5 μm) using 1% formic acid in water (phase A) and acetonitrile (phase B) as the mobile phases at a flow rate of 0.5 mL/min. The gradient program was as follows: 0 min, 5% B; 1–5 min, 15% B; 5–25 min, 25% B; 25–40 min, 55% B; 40–50 min, 95% B; and 50–60 min, 5% B. Mass spectra were recorded in negative ion mode over an *m*/*z* range of 150–2000. The ESI-qTOF-MS parameters included a capillary temperature of 220 °C, sheath gas at 30 A.U., and a source voltage of 4 kV. Compounds were identified by comparing retention times, *m/z* values, and fragment patterns with those of standards and literature data (Liu et al., 2025; Yang et al., 2019). Detailed identification parameters are provided in Supplementary Table S1. The contents of individual phenolic compounds were calculated and expressed as mg/kg dry weight (mg/kg DW).

### 2.14 Statistical analysis

All experiments were conducted in triplicate, and the results were expressed as mean ± standard deviation (SD). Statistical analyses were performed using SPSS software (version 17.0; SPSS Inc., Chicago, IL, USA). Data were analyzed by one-way analysis of variance (ANOVA), followed by Duncan's multiple range test for post hoc comparisons. Differences were considered statistically significant at *p* < 0.05. In addition, principal component analysis (PCA) was performed using R software to evaluate the overall variation among samples.

## 3 Results and discussion

### 3.1 Changes in texture properties

**Fig. 2:**
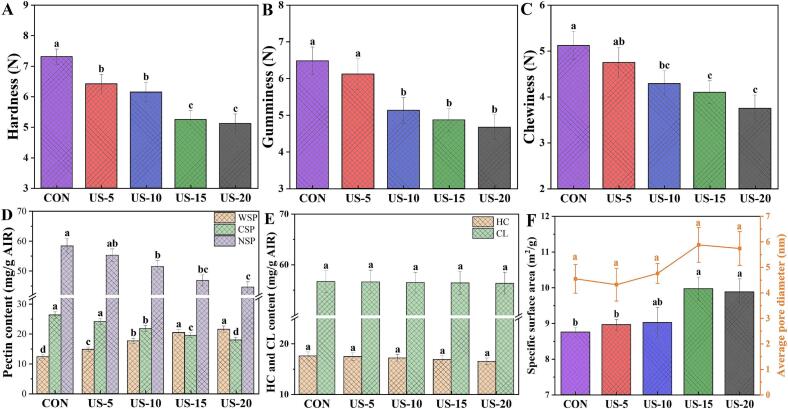

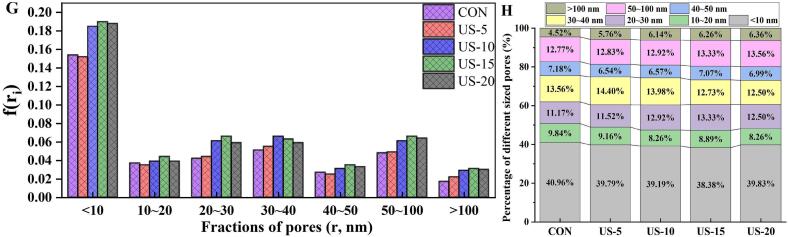
Effects of ultrasound treatment time on the physicochemical properties of raspberries. (A) Fruit hardness; (B) fruit gumminess; (C) fruit chewiness; (D) cell wall pectin content; (E) cell wall hemicellulose and cellulose contents; (F) specific surface area and average pore diameter; (G) pore size distribution; and (H) porosity. Different lowercase letters represent significant differences among the samples (*p* < 0.05).

As shown in Fig. 2A–C, ultrasound pretreatment caused a progressive softening of raspberry tissue. Hardness, gumminess, and chewiness all decreased as the ultrasound time increased from 0 to 20 min (p < 0.05). Hardness, which reflects the peak force during the first compression, decreased by about 30.0%, from 7.31 N in the control group to 5.12 N in the US-20 group. Gumminess and chewiness showed similar trends, decreasing from 6.48 to 4.67 N and from 5.12 to 3.75 N, respectively. This softening was most likely associated with ultrasound-induced disruption of the cell wall matrix and a reduction in cellular turgor.

Acoustic cavitation, microstreaming, and mechanical vibration can weaken intercellular adhesion and promote the disassembly of structural polysaccharides, especially pectin in the middle lamella. Because pectin is the main component responsible for cell-to-cell adhesion, its solubilization and depolymerization would be expected to reduce tissue firmness directly (Wang et al., 2023). It is possible that ultrasound promoted pectin solubilization and depolymerization, leading to the conversion of CSP and NSP pectin into more weakly bound WSP (Wang, Li, Xue, et al., 2024). As this process progressed, the pectin network may have gradually weakened, thereby reducing cell-to-cell adhesion and tissue firmness. This structural weakening likely accounts for the lower hardness, gumminess, and chewiness observed after ultrasound pretreatment.

### 3.2 Microstructure analysis

**Fig. 3:**
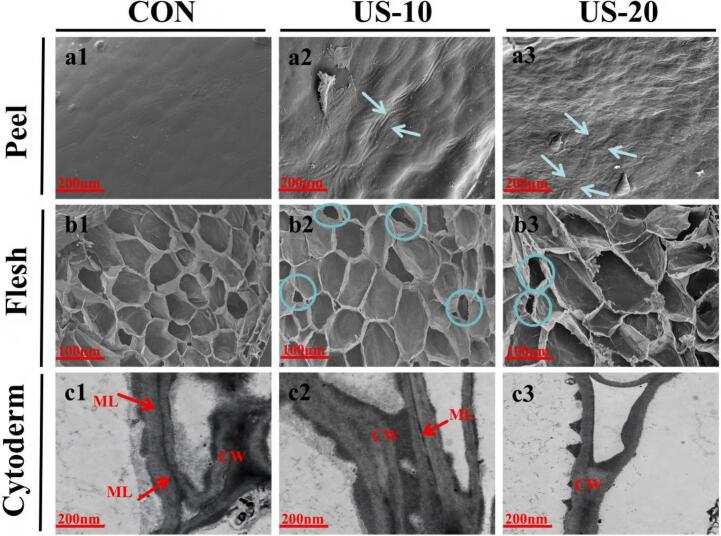
Microscopy images of raspberry tissues before and after ultrasonication. Note: First line: SEM images of peel (a1–a3), in second line-SEM images of flesh (b1–b3) and in third line-TEM images of cell wall (c1–c3); the first to third columns represent the different ultrasonic treatment times with 0, 10, and 20 min, respectively.

The integrity of the fruit cell wall plays an important role in regulating internal water distribution and permeability, and therefore strongly affects drying behavior (Miraei Ashtiani et al., 2020; Wang, Li, Xue, et al., 2024). To clarify the structural basis of tissue softening, SEM and TEM were used to examine raspberry tissues after ultrasound pretreatment, and representative images of the control, US-10, and US-20 groups are shown in Fig. 3. The peel of the untreated fruit exhibited a smooth and relatively intact surface, whereas ultrasound-treated samples gradually developed folds and wrinkles, particularly after 20 min of treatment. This change suggests that ultrasound progressively weakened the structural continuity of the fruit surface.

The flesh tissue of the control group showed a compact and well-organized honeycomb-like structure with closely connected and fully expanded cells. After ultrasound pretreatment, however, the tissue became progressively looser, with wrinkled cell walls and more evident intercellular voids. These features indicate that ultrasound reduced tissue compactness and created additional pathways for internal moisture movement. Similar structural loosening has been reported in chili peppers (Nian et al., 2024), apples (Wang et al., 2026), and mulberries (Wang, Li, Xue, et al., 2024), where the formation of pores and microchannels was considered beneficial for mass transfer.

As shown in Fig. 3c1–c3, TEM observations further revealed that the middle lamella in fresh raspberries was continuous and clearly distinguishable, whereas its boundary became blurred and partially discontinuous after ultrasound treatment. Because the middle lamella is rich in pectin, these ultrastructural changes strongly suggest degradation or transformation of pectic substances (Zhang et al., 2025). Importantly, these observations are consistent with the texture results above: the weakening of the middle lamella would reduce intercellular adhesion and thereby contribute to the decline in hardness, gumminess, and chewiness. At the same time, the emergence of a looser and more porous structure implies that the same structural damage that softened the tissues may also facilitate subsequent water migration during drying. It should also be noted that ultrasound-induced structural changes may not be limited to the cell wall. Acoustic cavitation, microstreaming, and mechanical vibration can also affect the integrity and permeability of cellular membranes (Dadan et al., 2025). Although membrane integrity was not directly measured in the present study, partial membrane disruption or increased membrane permeability may have occurred during ultrasound treatment, leading to intracellular leakage and reduced cellular turgor. This effect could further contribute to tissue softening and the formation of more continuous pathways for moisture migration.

### 3.3 Cell wall polysaccharide content

The contents of the WSP, CSP, NSP, HC, CL are shown in Fig. 2. With increasing ultrasound time, the content of cell-wall polysaccharides was changed, particularly in the pectin fractions. After 20 min of treatment, WSP increased from 12.35 to 21.54 mg/g AIR, whereas CSP and NSP significantly decreased from 26.33 to 17.96 and 58.38 to 44.61 mg/g AIR, respectively (*p* < 0.05). Among these fractions, NSP remained the dominant pectin fraction, but its continuous decrease indicates that ultrasound substantially affected the tightly associated pectin network.

These results suggest that ultrasound may have promoted the breakdown and solubilization of the pectin matrix. The mechanical action and cavitation generated during treatment may have disrupted the bonds that maintain CSP and NSP within the cell wall and middle lamella, resulting in their conversion into the more loosely bound water-soluble fraction (Yapias et al., 2025). This change in pectin content may partly explain the textural deterioration observed after ultrasound pretreatment. As the structural pectin network weakened, cell-to-cell adhesion may also have decreased, which could reduce the integrity of the middle lamella and contribute to the decline in hardness, gumminess, and chewiness. Correlation analysis revealed that WSP content was significantly negatively correlated with hardness, gumminess, and chewiness (p < 0.05, *r* > 0.98), whereas CSP and NSP contents were positively correlated with these textural parameters (p < 0.05, *r* > 0.91) (Fig.S1, Supplementary materials).

In contrast, HC exhibited only a slight decrease (from 17.56 to 16.51 mg/g AIR) primarily after prolonged treatment (*p* > 0.05), suggesting it is less sensitive to ultrasound than pectin. This resistance may be attributed to the fact that HC is composed of branched, heterogeneous polysaccharides deeply embedded within the cell wall matrix, possessing a structure that is often more crystalline and robust than the gel-like, easily disrupted pectin (Pękala et al., 2023). By comparison, CL content remained relatively stable across all treatments (p > 0.05), indicating that the CL microfibril network remained largely unaffected and continued to serve as the primary structural framework. In summary, ultrasound mainly disrupted the pectin matrix, which explains the significant texture softening, whereas the HC and CL components remained largely intact.

### 3.4 Porous structure of cell wall materials

As shown in Fig. 2F–H, ultrasound treatment modified the porous characteristics of raspberry cell walls. The SSA increased from 8.75 m^2^/g in the control to a maximum of 9.97 m^2^/g at US-15, followed by a slight decline to 9.88 m^2^/g at US-20 (Fig. 2F). By contrast, the average pore diameter (APD) only showed an increasing tendency with increasing ultrasound exposure time, rising from 4.55 nm in the control to 5.88 nm at US-15, but no statistically significant differences were observed among treatments (*p* > 0.05) (Fig. 2F). Previous studies have shown that the total pore surface area in plant tissue is primarily dictated by fine pores under 10 nm (Wen et al., 2021). In the present study, the pore size distribution (Fig. 2G) further showed that raspberry cell walls consisted predominantly of mesopores, with a smaller contribution from macropores. Although pores <10 nm remained the most frequent, their proportion decreased from 40.96% in the control to 38.38% at US-15, whereas the proportions of larger mesopores (20–50 nm) and macropores (>100 nm) showed an upward trend. These results indicate that ultrasound pretreatment reshaped the pore size distribution, possibly by promoting the expansion or connection of some fine pores into larger transport pathways. Such changes, together with the increased SSA, suggest that moderate ultrasonic treatment improved the accessibility and continuity of internal moisture migration channels, rather than simply enlarging the average pore diameter.

Interestingly, despite the shift toward larger pore channels, the calculated APD showed no statistically significant differences across treatments (p > 0.05). This result may be explained by the fact that APD is a weighted average parameter and therefore may mask simultaneous changes occurring in different pore-size ranges. During ultrasonic treatment, acoustic cavitation may promote the expansion and merging of some small pores into larger channels, while also inducing localized collapse of fragile cell wall regions or the formation of minute new voids (Sun et al., 2026). These concurrent and partially opposing effects may offset each other in the averaged APD value, ultimately leaving APD statistically unchanged while still reshaping the overall pore-size distribution. Therefore, the enhanced pore structure observed in this study is mainly reflected by the increased SSA and redistribution of pore sizes, rather than by a statistically significant increase in APD. These pore-structure results agree with the observed changes in microstructure and pectin fractions. As CSP and NSP were progressively solubilized and the middle lamella became discontinuous, the cell-wall network became less compact and more permeable, which would naturally increase accessible surface area and favor the formation of wider channels. Correlation analysis further confirmed these results, revealing that SSA and APD were significantly positively correlated with WSP content (*p* < 0.05, *r* > 0.89), whereas significant negative correlations were observed with CSP and NSP fractions (p < 0.05, *r* > 0.88) (Fig.S1, Supplementary materials). The fact that the SSA peaked at US-15 and then slightly declined at US-20 further suggests that excessive ultrasound did not continue to improve the porous structure indefinitely. Instead, the loosening effect appeared to level off after 15 min, and longer treatment may have caused partial collapse of fragile wall regions. Nevertheless, the possible influence of freeze-drying on pore-structure measurements should be considered. Freeze-drying may modify the native pore network of plant tissues, and thus the absolute SSA and pore-size values obtained by nitrogen adsorption may not fully represent the hydrated state of fresh raspberry cell walls. However, because all treatment groups were prepared using the same freeze-drying and analytical procedures, the comparison among groups remains meaningful. Therefore, the increase in SSA and the shift in pore-size distribution are interpreted as relative changes in the apparent porous structure of freeze-dried cell-wall materials caused by ultrasound pretreatment, rather than as direct quantitative measurements of native hydrated pores.

### 3.5 Mw and DE of pectin fraction

As shown in Table 1, the Mw of all three pectin fractions decreased after ultrasound treatment, although the extent of reduction differed among fractions. WSP exhibited the most pronounced decrease, with Mw dropping significantly at US-15 and US-20, indicating that this fraction was highly susceptible to ultrasound-induced depolymerization. CSP showed only a moderate decrease in Mw, with a significant decrease observed only in the US-20 group (*p* < 0.05). NSP was the most stable fraction, as its Mw remained largely unchanged under most treatment conditions and decreased significantly only at US-20. These results suggest that ultrasound promoted the degradation of pectin molecular chains through cavitation-induced cleavage of glycosidic linkages, but the effect was highly dependent on the binding state and structural compactness of each fraction (Xing et al., 2025). The more loosely bound nature of WSP may have made it more vulnerable to ultrasonic disruption, whereas the stronger association of CSP and NSP with the cell wall matrix likely contributed to their greater resistance to depolymerization (Gao et al., 2024). Furthermore, correlation analysis indicated that the Mw of WSP was significantly negatively correlated with WSP content, SSA, and APD (p < 0.05, *r* > 0.91), while showing significant positive correlations with hardness, chewiness, and the contents of CSP and NSP (p < 0.05, *r* > 0.82) (Fig.S1, Supplementary materials).

**Table 1:** The physicochemical properties of pectin samples.

Physico-chemical properties	WSP					CSP					NSP				
	CON	US-5	US-10	US-15	US-20	CON	US-5	US-10	US-15	US-20	CON	US-5	US-10	US-15	US-20
Mw (g/mol) × 10^5^	9.17 ± 0.31^a^	8.69 ± 0.37^a^	8.83 ± 0.38^a^	7.22 ± 0.18^b^	6.49 ± 0.25^c^	6.34 ± 0.23^a^	6.28 ± 0.18^a^	6.08 ± 0.21^ab^	6.12 ± 0.19^ab^	5.65 ± 0.15^b^	4.37 ± 0.11^a^	4.31 ± 0.13^a^	4.28 ± 0.12^a^	4.15 ± 0.12^a^	3.55 ± 0.13^b^
DE (%)	24.91 ± 0.13^b^	24.85 ± 0.17^b^	25.19 ± 0.11^ab^	25.86 ± 0.21^a^	25.13 ± 0.09^ab^	5.13 ± 0.11^a^	5.07 ± 0.13^a^	5.15 ± 0.15^a^	4.96 ± 0.17^a^	5.03 ± 0.15^a^	–	–	–	–	–
Man	1.75 ± 0.04^a^	1.73 ± 0.04^a^	0.96 ± 0.04^b^	0.86 ± 0.03^b^	0.92 ± 0.05^b^	0.68 ± 0.04^c^	0.66 ± 0.03^c^	0.85 ± 0.04^a^	0.88 ± 0.04^a^	0.79 ± 0.03^a^	0.28 ± 0.02^b^	0.31 ± 0.03^b^	0.33 ± 0.02^b^	0.57 ± 0.03^a^	0.62 ± 0.02^a^
Rha	5.15 ± 0.23^c^	5.57 ± 0.14^b^	5.88 ± 0.17^ab^	5.76 ± 0.13^ab^	6.07 ± 0.15^a^	9.98 ± 0.31^a^	8.75 ± 0.28^b^	8.65 ± 0.26^b^	8.69 ± 0.37^b^	8.55 ± 0.29^b^	11.85 ± 0.38^a^	11.67 ± 0.75^a^	11.39 ± 0.35^a^	11.55 ± 0.43^a^	11.67 ± 0.39^a^
GlcA	1.25 ± 0.04^a^	1.38 ± 0.03^a^	1.21 ± 0.04^a^	1.29 ± 0.04^a^	1.24 ± 0.03^a^	1.68 ± 0.04^b^	1.89 ± 0.04^a^	1.88 ± 0.03^a^	1.75 ± 0.05^ab^	1.65 ± 0.06^b^	1.75 ± 0.06^a^	1.72 ± 0.04^a^	1.55 ± 0.04^b^	1.37 ± 0.03^c^	1.53 ± 0.04^b^
GalA	28.66 ± 1.23^c^	32.58 ± 1.35^b^	35.75 ± 1.32^ab^	36.12 ± 1.33^a^	37.16 ± 1.57^a^	38.55 ± 1.21^a^	39.19 ± 1.08^a^	38.22 ± 1.13^a^	37.17 ± 1.21^a^	36.38 ± 1.17^a^	25.65 ± 0.83^a^	25.18 ± 0.72^ab^	23.33 ± 0.54^b^	25.39 ± 0.37^ab^	25.22 ± 0.38^ab^
Glc	7.96 ± 0.13^a^	7.88 ± 0.20^a^	6.28 ± 0.26^b^	6.17 ± 0.31^b^	6.20 ± 0.38^b^	6.13 ± 0.13^d^	6.85 ± 0.18^c^	7.23 ± 0.21^bc^	7.55 ± 0.20^b^	8.68 ± 0.19^a^	1.26 ± 0.04^c^	1.31 ± 0.05^c^	1.55 ± 0.06^b^	1.88 ± 0.07^a^	1.72 ± 0.06^a^
Gal	42.19 ± 1.76^a^	40.28 ± 1.88^a^	35.19 ± 1.25^b^	32.17 ± 1.13^bc^	30.82 ± 1.05^c^	18.55 ± 1.01^a^	18.32 ± 1.22^a^	17.65 ± 1.21^ab^	17.19 ± 1.14^ab^	15.58 ± 1.08^b^	25.18 ± 1.32^a^	26.37 ± 1.27^a^	26.33 ± 1.25^a^	25.73 ± 1.19^a^	25.83 ± 1.25^a^
Xyl	0.89 ± 0.03^b^	0.96 ± 0.04^b^	1.23 ± 0.04^a^	1.35 ± 0.03^a^	1.32 ± 0.05^a^	1.58 ± 0.03^b^	1.75 ± 0.03^a^	1.68 ± 0.05^ab^	1.53 ± 0.05^b^	1.61 ± 0.03^ab^	0.66 ± 0.03^b^	0.68 ± 0.04^b^	0.75 ± 0.03^ab^	0.83 ± 0.04^a^	0.73 ± 0.05^ab^
Ara	10.80 ± 1.21^bc^	8.54 ± 1.01^c^	12.65 ± 1.32^b^	15.45 ± 1.08^a^	15.39 ± 1.11^a^	21.58 ± 1.28^b^	21.04 ± 1.30^b^	22.37 ± 1.33^ab^	23.72 ± 1.23^ab^	25.10 ± 1.17^a^	32.41 ± 1.85^a^	31.91 ± 1.75^a^	33.89 ± 1.63^a^	31.96 ± 1.52^a^	31.87 ± 1.88^a^
Fuc	1.35 ± 0.02^a^	1.08 ± 0.03^b^	0.85 ± 0.03^c^	0.83 ± 0.04^c^	0.88 ± 0.06^c^	1.27 ± 0.03^c^	1.55 ± 0.04^ab^	1.47 ± 0.05^b^	1.52 ± 0.04^ab^	1.66 ± 0.03^a^	0.96 ± 0.03^a^	0.85 ± 0.05^ab^	0.88 ± 0.04^ab^	0.72 ± 0.03^b^	0.81 ± 0.04^ab^
R1	26.81 ± 0.88^c^	30.72 ± 0.76^b^	32.93 ± 0.89^ab^	33.42 ± 1.03^ab^	34.26 ± 1.11^a^	31.66 ± 0.81^a^	31.18 ± 0.95^a^	33.39 ± 0.85^a^	32.25 ± 0.82^a^	31.91 ± 0.95^a^	14.41 ± 0.68^a^	14.10 ± 0.73^a^	12.48 ± 0.85^b^	14.50 ± 1.63^a^	14.22 ± 0.45^a^
R2	72.17 ± 1.21^a^	68.19 ± 1.13^ab^	65.71 ± 1.22^b^	65.10 ± 1.11^b^	64.29 ± 1.36^b^	66.59 ± 1.29^a^	63.85 ± 1.76^a^	64.72 ± 1.75^a^	66.01 ± 1.81^a^	66.25 ± 1.55^a^	84.90 ± 1.21^a^	85.19 ± 1.72^a^	86.74 ± 1.35^a^	84.63 ± 1.37^a^	85.02 ± 1.53^a^
R3	10.29 ± 0.73^a^	8.76 ± 0.40^b^	8.14 ± 0.15^bc^	8.27 ± 0.16^bc^	7.61 ± 0.23^c^	4.02 ± 0.21^b^	4.50 ± 0.18^ab^	4.63 ± 0.15^ab^	4.71 ± 0.13^a^	4.76 ± 0.15^a^	4.86 ± 0.20^a^	4.99 ± 0.11^a^	5.29 ± 0.21^a^	4.99 ± 0.15^a^	4.94 ± 0.18^a^

Note: Distinct letters represent significant differences between various ultrasonic times (p < 0.05). Man, mannose; Rha, rhamnose; GlcA, glucuronic acid; GalA, galacturonic acid; Glc, glucose; Gal, galactose; Xyl, xylose; Ara, arabinose; Fuc, fucose. R1 (mol%) = (GalA-Rha)/(GalA + Rha + Ara + Gal + Xyl) × 100; R2 (mol%) = (2Rha + Ara + Gal)/(GalA + Rha + Ara + Gal + Xyl) × 100; R3 = (Gal +Ara)/Rha.

As shown in Table 1, the DE values of both WSP and CSP were lower than 50%, indicating that both fractions belonged to low-methyl ester pectin (Xing et al., 2025). The DE of WSP increased slightly, reaching a significant level only at US-15 (p < 0.05), while the DE of CSP remained statistically unchanged (*p* > 0.05). In general, the DE of CSP was consistently much lower than that of WSP, which may be related to its binding state in fruit tissue. CSP is mainly associated with the cell wall through Ca^2+^ mediated cross-linking between unesterified GalA residues and is therefore more tightly retained within the cell wall matrix, resulting in a lower DE than WSP (Sun et al., 2023). The slight increase in DE of WSP after ultrasound treatment is more likely attributable to structural rearrangement and the preferential solubilization or enrichment of pectin domains with relatively higher methyl esterification, rather than to direct ultrasound-induced esterification (Xing et al., 2025). Previous studies have reported that ultrasonic treatment generally leads to a decrease in the DE of extracted pectin (Zhang et al., 2022). In the present study, pectin was extracted after ultrasonic treatment of the raw material, and the DE did not show an obvious decreasing trend, suggesting that the effect of ultrasound on methyl ester groups may depend on the pectin source and processing mode (Xing et al., 2025). Besides, The DE of NSP was not determined because its ester bonds were cleaved during the extraction process.

### 3.6 Monosaccharide composition of pectin fraction

The monosaccharide composition further indicated that ultrasound caused changes in the pectin structure of raspberry (Table 1). GalA is mainly derived from homogalacturonan (HG), whereas rhamnose (Rha) together with arabinose (Ara) and galactose (Gal) is generally associated with the backbone and side chains of rhamnogalacturonan-I (RG-I) (Xing et al., 2025). Therefore, changes in monosaccharide composition can reflect variations in the relative distribution of linear and branched pectic domains. Among the three fractions, WSP showed the largest compositional changes. With increasing ultrasonic time, GalA increased significantly from 28.66% to 37.16%, whereas Gal decreased from 42.19% to 30.82%. Mannose (Man) and fucose (Fuc) also decreased significantly, while Ara increased overall, especially at US-15 and US-20. These results suggest that ultrasound increased the relative proportion of the GalA-rich backbone domain in WSP while reducing neutral sugar-rich side chains, particularly galactan-rich branches. The decrease in Gal together with the increase in GalA suggests that RG-I side chains were more susceptible to ultrasonic degradation than the uronic acid-rich backbone (Wang, Li, Xue, et al., 2024). The increase in Ara may reflect a relative enrichment of arabinan residues. Combined with previous reports showing that ultrasonic treatment disrupts pectin branching and modifies the RG-I region (Wang, Li, Xue, et al., 2024), this observation may indicate that Ara-containing side chains were less affected than galactan-rich branches under the present conditions. Similar results were also reported by Xing et al. (2025) during the ultrasonic treatment of strawberry pulp, possibly because acoustic cavitation preferentially degraded the more exposed galactan-rich branches, whereas Ara-containing side chains were relatively less affected, leading to an apparent increase in Ara content.

By contrast, CSP exhibited only minor changes in monosaccharide composition after ultrasound treatment. GalA remained the predominant monosaccharide, with no significant difference (*p* > 0.05) in its relative proportion among treatments. The relative proportion of Rha decreased slightly, while Ara showed an increase from 21.58% to 25.10%. Meanwhile, the relative proportion of glucose (Glc) gradually increased, whereas that of Gal decreased slightly after prolonged ultrasound. These results indicate that, unlike WSP, the overall distribution of structural domains in CSP remained relatively stable under the applied ultrasonic conditions, although slight changes of side chains may still have occurred. The relative proportion of GalA in NSP decreased slightly at US-10, whereas those of Ara, Gal, and Rha remained largely unchanged throughout the treatments, suggesting that NSP maintained a relatively rigid and stable structure during ultrasonic treatment, probably owing to its stronger association with the cell wall matrix (Gao et al., 2024).

To better describe the structural changes beyond individual monosaccharides, the calculated indices R1, R2, and R3 were used to estimate the relative contribution of HG rich regions, RG-I rich regions, and side-chain development. The molar proportions of the HG region in all three raspberry pectin fractions were below 50%, indicating that RG-I was the predominant structural domain (Xing et al., 2025). In WSP, R1 increased significantly from 26.81% to 34.26% with increasing ultrasonic time, whereas R2 decreased from 72.17% to 64.29% and R3 decreased from 10.29% to 7.61%. This result supports that ultrasound reduced the relative abundance of RG-I side chains in WSP and increased the proportion of linear HG domains. The decrease in R3 also indicates shortening of galactan branches from the RG-I backbone, suggesting that ultrasound promoted side-chain cleavage and resulted in a less branched WSP structure. In comparison, both CSP and NSP exhibited only slight changes in their structural indices during ultrasound treatment. Although CSP showed a minor increase in R3, the overall structures of both fractions remained relatively stable.

Overall, ultrasound had the greatest effect on WSP, as reflected by an increase in HG-like regions and a decrease in Gal-rich side chains. In contrast, CSP and NSP showed only limited changes, which may be related to their different binding states. CSP and NSP are more tightly bound to the cell wall through ionic and covalent interactions, whereas the more loosely bound or free state of WSP may make it more susceptible to ultrasound-induced structural modification (Gao et al., 2024).

### 3.7 Water distribution state analysis

**Fig. 4:**
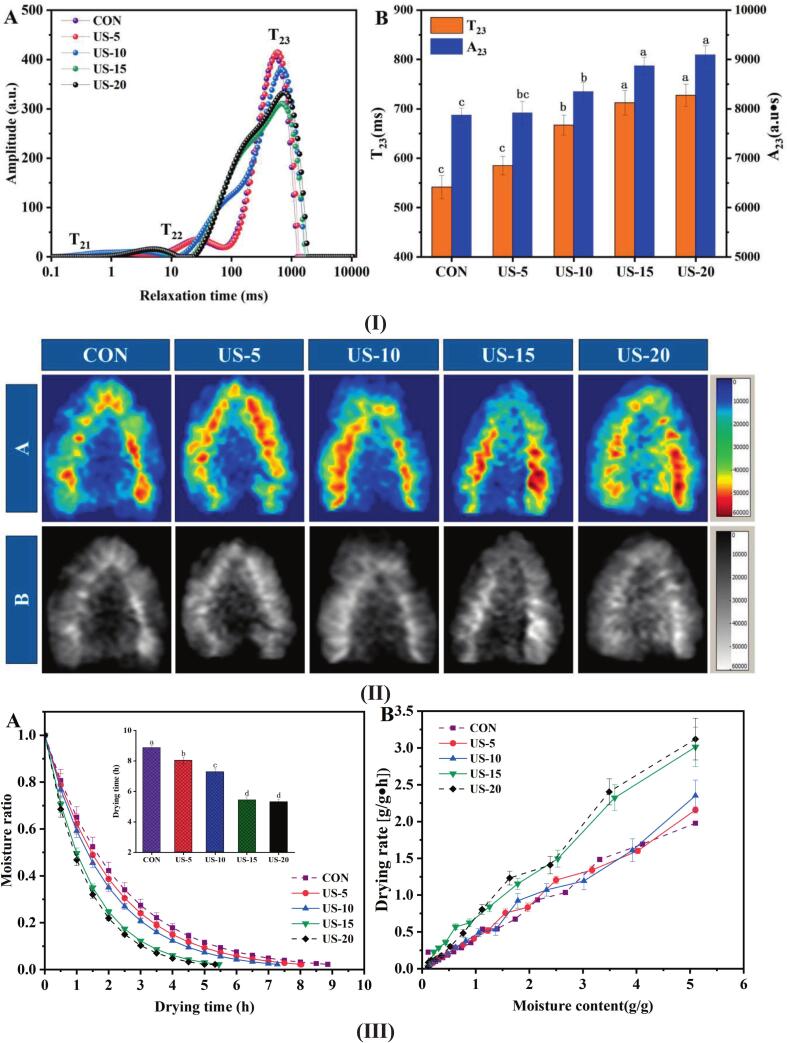
Carr-Purcell-Meiboom-Gill (CPMG) proton distribution profiles (A), T_2_ relaxation time distributions and corresponding relative peak areas derived from CPMG analysis (B) (I), proton density-weighted T_2_ images (II), as well as the relationships of moisture ratio with drying time (A) and drying rate with moisture content (B) (III) for raspberries before and after ultrasonic pretreatment.

LF-NMR and MRI were used to investigate the alterations in the state and distribution of water within raspberry tissues induced by ultrasound treatment. As shown in Fig. 4I-A, the transverse relaxation curves of all samples could be decomposed into three components, corresponding to tightly bound water (T₂₁), less mobile water in the cytoplasmic region (T₂₂) and freely moving water located mainly in vacuoles and intercellular spaces (T₂₃). Among these, the T₂₃ component was the most prominent in terms of signal intensity, indicating that free water was the predominant form of moisture in fresh raspberries. To better evaluate the effect of ultrasonication on free water, the relaxation time (T₂₃) and corresponding peak area (A₂₃) were quantified (Fig. 4I-B). In fresh fruits, the T₂₃ and A₂₃ were 541.58 ms and 7869.33 a.u., respectively. Both parameters gradually increased as the ultrasonication time increased, reaching 712.38 ms and 8869.37 a.u., respectively, after 15 min of treatment. When the ultrasonication time was further increased to 20 min, T₂₃ and A₂₃ showed no significant differences compared with those at 15 min (*p* > 0.05). This result suggests that the effect of ultrasonication on water mobility approached a plateau after 15 min. Studies have demonstrated that an elongation of the T₂ relaxation time is generally associated with weaker interactions between water molecules and the surrounding matrix, thereby reflecting higher molecular mobility of H protons (Wang, Li, He, et al., 2024). In conjunction with the degradation of CSP and NSP, the increases in water mobility and free water proportion observed in this study may be explained by the cavitation effect induced by ultrasonication, which weakened the association between water molecules and cell wall pectin, thereby facilitating water migration into a freer state. Correlation analysis further confirmed these findings, revealing that T_23_ was positively correlated with WSP content and its R1 ratio (*p* < 0.05, *r* > 0.88), while being negatively correlated with CSP and NSP contents as well as the R2 and R3 ratio of WSP (p < 0.05, *r* > 0.96). Similarly, A_23_ showed a positive correlation with WSP content (p < 0.05, *r* = 0.97) but significant negative correlations with CSP and NSP contents and the Mw of WSP (p < 0.05, *r* > 0.94).

The MRI images provide spatial evidence for these changes (Fig. 4II). In the fresh raspberries, proton density was relatively uniform and moderate, with only a limited region of stronger signal near the central pulp. After ultrasound treatment, high-intensity areas expanded and became more pronounced, particularly in samples treated for 15 and 20 min, where bright regions spread from the inner tissues toward the periphery. This changes in signal intensity imply that free water accumulated and redistributed within the flesh as the treatment time increased. Combining these observations with our structural results (Table 1), it can be inferred that ultrasound disrupts the side chains of cell wall polysaccharides and loosens the pectin network, thereby weakening the binding sites that previously immobilized water. As a consequence, part of the bound and weakly bound water is released into larger pores and intercellular spaces, giving rise to longer T₂₃ and higher A₂₃. Among the tested ultrasound durations, US-15 was the most suitable treatment for increasing water mobility and improving the continuity of moisture pathways in raspberry tissues, which is expected to facilitate moisture migration during subsequent drying and influence the quality of the dried product.

### 3.8 Drying characteristics

Drying is an effective approach for extending the shelf life of raspberries while maintaining their quality. As shown in Fig. 4III-A, ultrasound pretreatment significantly shortened the drying time of raspberries compared with the control group (CON). Specifically, the drying time decreased from 8.87 h in CON to 5.53 h in the US-15 group and 5.33 h in the US-20 group, corresponding to reductions of 37.7% and 39.9%, respectively. Consistently, the ultrasound-treated groups exhibited higher drying rates than CON, with the highest drying rate observed in the US-15 and US-20 groups (Fig. 4III-B). However, the drying time of the US-15 group was only 0.20 h longer than that of the US-20 group, and no significant difference was detected between the two treatments (*p* > 0.05), suggesting that the promoting effect of ultrasonication on drying gradually approached a plateau after 15 min rather than increasing indefinitely with treatment time. This improvement in drying behavior may be attributed to the ultrasound-induced disruption of the cell wall matrix, increased tissue porosity, and enhanced water mobility, which collectively reduced internal resistance to moisture migration and facilitated water removal during drying (Wang, Li, Xue, et al., 2024). Similar promoting effects of ultrasound on drying kinetics have been widely reported in fruit tissues, although the influence of ultrasound conditions is parameter-dependent (Zhang et al., 2025; Zhou et al., 2024). Furthermore, correlation analysis indicated that the drying time of raspberries was positively correlated with CSP and NSP contents, as well as the Mw and R2 of WSP (*p* < 0.05, *r* > 0.90). Conversely, significant negative correlations were observed between drying time and SSA, APD, WSP content, the R1 of WSP, as well as T_23_ and A_23_ (p < 0.05, *r* > 0.89). The mechanism of ultrasound-enhanced raspberry drying is proposed as follows: acoustic cavitation triggers the degradation of pectin side chains and the solubilization of CSP and NSP, thereby increasing cell wall porosity and specific surface area. These modifications weaken the interaction between water and the cellular matrix, liberating bound water and increasing its mobility. This shift in water status establishes efficient pathways for moisture migration, significantly reducing drying time. Moreover, previous studies have indicated that excessive processing may adversely affect quality attributes, including phenolic retention, due to excessive ultrasound (Wang, Li, Xue, et al., 2024). Therefore, appropriate ultrasonication conditions are essential to achieve a balance between drying efficiency and product quality.

### 3.9 Individual phenolics content

Phenolics are major bioactive components in raspberries, and their retention is an important criterion for assessing the effectiveness of drying conditions (Jaouhari et al., 2025). The quantitative analysis revealed simultaneous effects of thermal degradation, matrix release, and sonochemical action (Table 2). A total of 15 phenolics were identified in raspberries, including five anthocyanins, three phenolic acids, three flavan-3-ols/proanthocyanidins, and four flavonols and their derivatives.

**Table 2:** Changes in phenolics content of raspberries before and after drying under different ultrasound conditions (mg/kg DW).

No.	Identification	Fresh	CON	US-5	US-10	US-15	US-20
1	Chlorogenic acid	13.52 ± 0.55^a^	8.27 ± 0.42^c^	8.69 ± 0.44^bc^	8.57 ± 0.52^bc^	9.65 ± 0.51^b^	9.32 ± 0.39^b^
2	(+)-Catechin	22.54 ± 1.63^b^	35.56 ± 2.39^a^	37.68 ± 1.96^a^	38.55 ± 1.78^a^	38.48 ± 1.83^a^	39.32 ± 2.05^a^
3	Brevifolin carboxylic acid	3.44 ± 0.12^b^	3.59 ± 0.10^b^	3.77 ± 0.15^b^	4.85 ± 0.18^ab^	5.67 ± 0.17^a^	5.88 ± 0.18^a^
4	Proanthocyanidin B1	624.70 ± 25.67^a^	475.18 ± 18.96^c^	486.32 ± 17.35^bc^	498.71 ± 17.32^bc^	542.18 ± 18.45^b^	550.23 ± 21.27^b^
5	(−)-Epicatechin	550.19 ± 14.33^a^	429.66 ± 20.18^b^	418.61 ± 18.36^b^	437.23 ± 15.73^b^	427.65 ± 20.61^b^	425.39 ± 18.56^b^
6	Ellagic acid	200.18 ± 8.55^b^	218.18 ± 8.62^b^	245.66 ± 7.39^ab^	265.83 ± 10.21^a^	270.33 ± 9.63^a^	267.93 ± 9.16^a^
7	Quercetin-3-glucuronide	485.49 ± 20.37^a^	407.52 ± 18.55^b^	418.55 ± 17.38^b^	427.56 ± 18.66^b^	425.55 ± 18.31^b^	437.38 ± 17.59^b^
8	Quercetin-3-rutinoside	147.42 ± 4.39^a^	138.55 ± 5.37^a^	142.76 ± 6.37^a^	140.28 ± 4.28^a^	150.33 ± 4.87^a^	151.52 ± 5.15^a^
9	Kaempferol-3-glucoside	17.52 ± 0.52^a^	16.86 ± 0.41^a^	17.28 ± 0.39^a^	17.89 ± 0.42^a^	17.16 ± 0.39^a^	16.37 ± 0.57^a^
10	Cyanidin-3-sophoroside	1995.53 ± 38.31^a^	1175.38 ± 35.33^d^	1287.36 ± 28.84^c^	1357.89 ± 31.27^bc^	1475.33 ± 28.95^b^	1235.38 ± 30.51^cd^
11	Quercetin	13.79 ± 0.53^c^	25.36 ± 0.43^b^	28.66 ± 0.47^a^	25.79 ± 0.39^b^	28.57 ± 0.46^a^	25.33 ± 0.51^b^
12	Cyanidin-3-glucoside	2386.10 ± 44.22^a^	1136.13 ± 35.39^d^	1257.38 ± 37.19^c^	1367.29 ± 37.22^b^	1388.96 ± 28.93^b^	1231.39 ± 26.19^c^
13	Cyanidin-3-glucosyl rutinoside	3775.22 ± 57.86^a^	2866.13 ± 63.74^bc^	2897.55 ± 61.22^bc^	2931.57 ± 60.58^bc^	3108.62 ± 48.92^b^	2826.37 ± 50.19^c^
14	Cyanidin-3-rutinoside	6585.10 ± 88.91^a^	3896.22 ± 75.33^cd^	3725.69 ± 68.37^d^	4019.33 ± 82.62^cd^	4439.61 ± 79.32^b^	3618.62 ± 85.19^d^
15	Pelargonidin-3-glucoside	98.90 ± 5.31^a^	37.85 ± 3.21^b^	36.55 ± 4.32^b^	38.16 ± 3.72^b^	45.94 ± 2.31^b^	42.34 ± 4.22^b^

Note: Different lowercase letters within the same row indicate significant differences among treatments (p < 0.05).

Anthocyanins were the most heat-sensitive phenolics during drying. In the CON group, cyanidin-3-sophoroside and cyanidin-3-glucoside decreased by approximately 41% and 52%, respectively, compared with fresh fruit, confirming the pronounced susceptibility of anthocyanins to prolonged thermal exposure. This degradation is mainly attributed to heat-induced nucleophilic attack of water on the flavylium cation, followed by ring opening and chalcone formation (Enaru et al., 2021). Ultrasound pretreatment for 15 min significantly alleviated these losses, with cyanidin-3-sophoroside and cyanidin-3-glucoside reaching 1475.33 and 1388.96 mg/kg, respectively, which were 25.5% and 22.3% higher than those in CON. A similar trend was also observed for cyanidin-3-glucosyl rutinoside and cyanidin-3-rutinoside, whose contents increased from 2866.13 and 3896.22 mg/kg in CON to 3108.62 and 4439.61 mg/kg in US-15, respectively, indicating that moderate ultrasound pretreatment exerted a protective effect on anthocyanins. This improvement is likely related to ultrasound-induced microstructural modification, including the formation of microchannels and loosening of the cell wall matrix, which enhanced effective moisture diffusivity and shortened thermal exposure during drying (Wang, Li, Xue, et al., 2024). However, when the ultrasound time was extended to 20 min, the retention of these anthocyanins declined again. In particular, cyanidin-3-glucosyl rutinoside and cyanidin-3-rutinoside decreased to 2826.37 and 3618.62 mg/kg, respectively, while cyanidin-3-sophoroside and cyanidin-3-glucoside also dropped significantly relative to US-15 (*p* < 0.05). This result suggests that excessive ultrasound may weaken the protective effect, probably because intensified cavitation and possible hydroxyl radical generation accelerate anthocyanin degradation (Mane et al., 2015).

In contrast to the degradation of anthocyanins, hydrolyzable tannins and their derivatives showed a distinct increasing trend. Interestingly, the remodeling of the cell wall's micro-porous structure, characterized by an increased SSA and expanded pore channels (Fig. 2F), provides a critical physical basis for the observed shifts in the phenolics. Consistently, the concentration of ellagic acid increased in all drying groups compared with fresh fruit, with the most significant elevations observed in the ultrasound-pretreated groups. This increase may be attributed to the breakdown of ellagitannin-related structures and the hydrothermal conversion of bound phenolic forms into free ellagic acid during drying (Teslić et al., 2022). The ultrasound-induced loosening of the cell wall matrix likely enhanced the accessibility of the plant tissue, thereby further facilitating these release and conversion processes (Wang, Li, Xue, et al., 2024). Meanwhile, brevifolin carboxylic acid increased progressively from 3.44 mg/kg in fresh fruit to 5.88 mg/kg in the US-20 group, indicating that prolonged ultrasound may also promote the oxidative transformation of certain tannin-related compounds. Overall, moderate ultrasound favored the release or preservation of some phenolics, whereas prolonged treatment appeared to promote further degradation or transformation. Furthermore, the data suggest the occurrence of thermal epimerization among flavan-3-ols during drying. A clear interconversion was observed between (−)-epicatechin and (+)-catechin; while epicatechin levels decreased in all dried samples, catechin levels nearly doubled compared to fresh fruit (reaching 39.32 mg/kg in US-20). This phenomenon suggests that thermal exposure facilitates the conversion of cis-epicatechin into its trans-epimer (catechin) (Fan et al., 2016). Additionally, the higher content of proanthocyanidin B1 in the ultrasound-treated samples, compared with its substantial loss in the CON group, indicates that ultrasound-induced cavitation has promoted cell wall disruption and facilitated the release of bound proanthocyanidins from the cell wall matrix, thereby mitigating the thermal degradation observed in the CON group. Overall, these results suggest that US-15 may be a relatively suitable treatment condition, as it balanced the preservation of thermolabile anthocyanins with the release of bound phenolics before oxidative degradation became more evident.

The interpretation of increased phenolic contents after drying requires consideration of several concurrent mechanisms. Because all phenolic contents were expressed on a dry-weight basis, the differences observed among treatments were not simply caused by moisture removal and concentration. For thermolabile anthocyanins, higher contents in ultrasound-pretreated samples relative to CON mainly reflected improved relative retention associated with reduced thermal exposure. In contrast, increases in ellagic acid and other tannin-related compounds may have resulted from enhanced extractability following cell-wall disruption, release of matrix-associated phenolics, and conversion of ellagitannin-related or bound forms during drying. Meanwhile, the opposite changes in (−)-epicatechin and (+)-catechin suggest that thermal transformation reactions, including epimerization, also contributed to the altered phenolic profile. Therefore, the observed changes in individual phenolics should be interpreted as the combined outcome of degradation, relative preservation, enhanced extractability, matrix release, and chemical transformation, rather than as a uniform preservation effect.

**Fig. 5:**
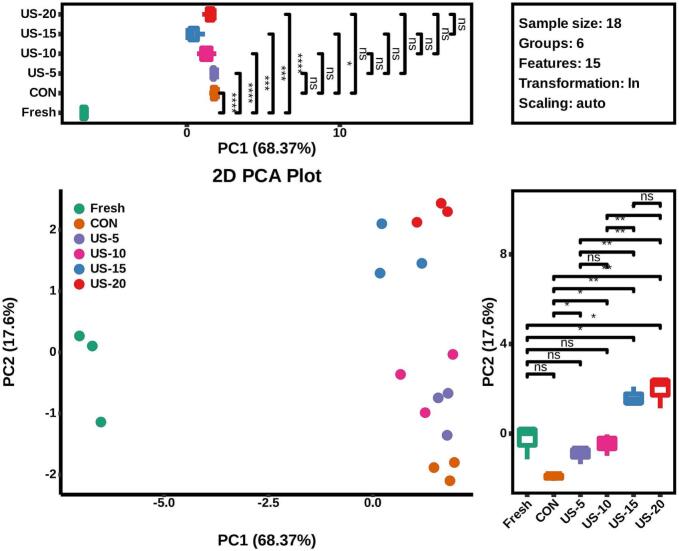
Principal component analysis of phenolics in raspberry samples before and after drying with different ultrasound pretreatments.

As shown in Fig. 5, PCA was performed on the quantified phenolic fingerprint (15 variables) of raspberry samples from six groups, using autoscaling without additional data transformation. PCA revealed a clear separation among raspberry samples subjected to different ultrasound pretreatments based on their phenolic profiles. PC1 and PC2 explained 68.37% and 17.60% of the total variance, respectively, accounting for 85.97% of the overall variability. Fresh samples were clearly separated from all dried samples along PC1, indicating that drying was the dominant factor driving the global shift in phenolic composition. Among the dried groups, CON, US-5, US-10, US-15, and US-20 showed a progressive redistribution mainly along PC2, suggesting a treatment-time-dependent remodeling of the phenolic profile induced by ultrasound. In particular, US-15 and US-20 were distinctly separated from CON and the shorter ultrasound treatments, indicating that moderate-to-prolonged sonication caused more pronounced changes in individual phenolics. The relatively tight clustering of biological replicates within each group further demonstrated good analytical reproducibility. Combined with the quantitative results of individual phenolics, the PCA supports that ultrasound pretreatment not only altered the overall phenolic composition of dried raspberries, but that a moderate treatment (US-15) provided a more favorable phenolic profile than excessive sonication.

### 3.10 Mechanistic insights and practical limitations

The present results suggested that ultrasound pretreatment improved raspberry heat pump drying mainly through matrix-level structural modification rather than simple cavitation-induced tissue disruption. Ultrasound preferentially remodeled the pectin network, as shown by increased WSP, decreased CSP and NSP, reduced pectin molecular weight, and relatively stable cellulose content. These changes loosened the middle lamella and cell-wall matrix, improved pore accessibility, and promoted water migration, which was further supported by LF-NMR and MRI results showing increased water mobility. Consequently, the shortened drying time could be partly attributed to reduced internal resistance to moisture transfer. This mechanism also contributed to phenolic retention by reducing the thermal exposure of anthocyanins and facilitating the release or conversion of ellagitannin-derived phenolics. Compared with previous studies focusing mainly on drying kinetics or bulk quality, this study links pectin transformation, water redistribution, and individual phenolic responses in the same heat pump drying system. Moreover, US-15 appeared to provide a better balance between drying efficiency and phenolic preservation, whereas prolonged ultrasound offered limited additional drying benefits and tended to reduce anthocyanin retention.

Nevertheless, several practical limitations should be considered before industrial application. Although ultrasound effectively modified raspberry tissue structure under laboratory-scale conditions, its scalability to large-batch or continuous processing remains to be verified. In particular, achieving uniform acoustic energy distribution in large volumes of fruit materials may be challenging, which could result in uneven pretreatment effects. In addition, ultrasound pretreatment requires extra energy input and equipment investment before drying; therefore, the reduction in drying time should be assessed together with the total energy consumption and economic cost of the whole process. Future studies should therefore focus on pilot-scale validation, energy-efficiency assessment, treatment uniformity, and cost-effectiveness analysis to determine the practical applicability of ultrasound pretreatment in commercial raspberry drying.

## 4 Conclusion

This study showed that ultrasound pretreatment improved heat pump drying of raspberries by altering cell wall structure and redistributing water within the tissue. Ultrasound mainly acted on the pectin matrix rather than the cellulose framework, promoting the conversion of tightly bound pectin fractions into WSP, decreasing pectin molecular weight, weakening side-chain branching, and loosening the middle lamella and cell wall network. These structural modifications increased the accessible surface area and altered the pore size distribution of the cell wall, thereby weakening the interaction between water molecules and the surrounding matrix and facilitating the transformation of immobilized water into more mobile water. Consequently, internal moisture transfer resistance was reduced and drying efficiency was markedly improved. Among the tested treatments, 15 min ultrasound pretreatment provided the most favorable balance between drying efficiency and product quality, shortening drying time by 39.9% while improving the retention of main anthocyanins and promoting the release of ellagitannin-derived phenolics. By contrast, US-20 resulted in only a marginal further shortening of drying time and tended to compromise the retention of thermolabile phenolic compounds. Therefore, moderate ultrasound pretreatment (US-15) can be considered an effective strategy for shortening drying time and enhancing phenolics content in heat pump-dried raspberries. These findings also provide new mechanistic insight into the relationship among pectin remodeling, water redistribution, and phenolics retention during fruit drying.

## CRediT authorship contribution statement

**Yi Li:** Writing – original draft, Software, Investigation, Conceptualization. **Simeng Wen:** Validation, Formal analysis, Data curation. **Xilei Sun:** Visualization, Validation. **Junjie Chen:** Methodology, Data curation. **qiaoyu Yang:** Supervision, Methodology, Data curation. **Jiahe Dai:** Writing – review & editing, Data curation. **Lijing Liu:** Data curation. **Hong Li:** Writing – review & editing, Supervision, Funding acquisition. **Kunhua Wang:** Writing – review & editing, Funding acquisition.

## Declaration of competing interest

The authors declare that they have no known competing financial interests or personal relationships that could have appeared to influence the work reported in this paper.

## Data Availability

No data was used for the research described in the article.
