# Supplementary material for: Ultrasound pretreatment enhances drying efficiency and phenolic retention in raspberries during heat pump drying by modulating cell wall structure and water status
Source: Food Chem X. 2026 Jul 14;38:104213. doi: 10.1016/j.fochx.2026.104213 (PMC13400451; doi:10.1016/j.fochx.2026.104213)
Supplement: Supplementary file 1 — Supplementary material. [file mmc1.docx]

**Supplementary Methods：**

**2.1 Plant material**

Total soluble solids (TSS) were measured at 25 °C using a digital handheld refractometer (PAL-1, Atago, Japan) and expressed as °Brix.

The initial moisture content was determined using a halogen moisture analyzer (SN-DHS-20A, SUNNE, China). Approximately 5.00 g of homogenized raspberry pulp was evenly spread on the sample pan and dried at 105 °C until the automatic endpoint was reached.

**2.10 Low field nuclear magnetic resonance (LF-NMR) and magnetic resonance imaging (MRI) analysis**

The CPMG echo decay curves were processed using the LF-NMR analysis software supplied with the Niumag system. The T₂ relaxation-time distribution was obtained by multi-exponential inversion of the decay signal, which was expressed as a sum of exponential components:

$$M\text{（}t\text{）=}\sum_{\text{i}} A_{i}\exp(-t/T_{2i})+\in$$

where *A_i_* represents the signal amplitude corresponding to each relaxation component, *T_2i_* is the transverse relaxation time, and ϵ represents residual noise.

Because the absolute T₂ ranges of different water populations may vary with sample characteristics and acquisition conditions, the water populations in this study were assigned according to the resolved peaks in the T₂ distribution rather than predefined universal relaxation-time intervals. The component with the shortest relaxation time was assigned to tightly bound water associated with cell-wall polysaccharides and macromolecules (T₂₁), the intermediate component to less mobile water mainly located in the cytoplasmic region (T₂₂), and the component with the longest relaxation time to free water mainly located in vacuoles and intercellular spaces (T₂₃). The boundaries between adjacent components were determined from the valleys between neighboring peaks in the T₂ distribution and were kept constant for all samples. The corresponding peak areas were calculated by integrating the signal intensity within each assigned T₂ region.

**Table S1** Qualitative analysis of 15 phenolics in raspberries.

| No. | RT (min) | Identification | Molecular formula | MS  [H-M]^-^/[H-M]^+^ | MS/MS | Standard curves  (μg/mL) | R^2^ |
| --- | --- | --- | --- | --- | --- | --- | --- |
| 1 | 2.45 | Chlorogenic acid | C_16_H_18_O_9_ | 353.09 | 191.00 | Y=12.82X-0.03 | 0.9985 |
| 2 | 2.61 | (+)-Catechin | C_15_H_14_O_6_ | 289.07 | 245.08，205.05，125.02 | Y=19.23X-2.21 | 0.9991 |
| 3 | 2.71 | Brevifolin carboxylic acid | C_13_H_8_O_8_ | 291.01 | 247.02 | Y=20.82X+0.82 | 0.9983 |
| 4 | 2.79 | Proanthocyanidin B1 | C_30_H_26_O_12_ | 577.14 | 407.08，289.07，245.08，205.05，125.02 | Y=4.93X-1.53 | 0.9987 |
| 5 | 3.90 | (-)-Epicatechin | C_15_H_14_O_6_ | 289.07 | 245.08，205.05，125.02 | Y=16.25X-4.22 | 0.9992 |
| 6 | 5.33 | Ellagic acid | C_14_H_6_O_8_ | 300.99 | 285.00 | Y=12.53X-0.02 | 0.9919 |
| 7 | 6.36 | Quercetin-3-glucuronide | C_21_H_18_O_13_ | 477.07 | 301.04，179.00，151.00 | Y=14.88X-3.30 | 0.9991 |
| 8 | 6.59 | Quercetin-3-rutinoside | C_27_H_30_O_16_ | 609.11 | 301.04，179.00，151.00 | Y=9.42X-4.72 | 0.9988 |
| 9 | 7.22 | Kaempferol-3-glucoside | C_21_H_20_O_11_ | 447.09 | 285.04 | Y=20.09X+1.38 | 0.9979 |
| 10 | 8.23 | Cyanidin-3-sophoroside | C_27_H_31_O_16_^+^ | 611.16^+^ | 287.05 | Y=9.49X +0.39 | 0.9992 |
| 11 | 9.40 | Quercetin | C_15_H_10_O_7_ | 301.04 | 179.00，151.00 | Y=70.18X -3.57 | 0.9992 |
| 12 | 9.61 | Cyaniding-3-glucoside | C_21_H_21_O_11_^+^ | 449.11^+^ | 287.05 | Y=8.88X +0.56 | 0.9989 |
| 13 | 10.39 | Cyanidin-3-glucosyl rutinoside | C_33_H_41_O_20_^+^ | 757.22^+^ | 287.05 | Y=9.32X+0.50 | 0.9987 |
| 14 | 12.03 | Cyanidin-3-rutinoside | C_27_H_31_O_15_^+^ | 595.16^+^ | 287.05 | Y=9.16X+0.43 | 0.9991 |
| 15 | 12.85 | Pelargonidin-3-glucoside | C_21_H_21_O_10_^+^ | 433.11^+^ | 271.06 | Y=9.09X+0.03 | 0.9992 |

**Fig.S1** Correlation analysis among physicochemical properties of raspberries.

Note:WSP, water-soluble pectin; CSP, chelate-soluble pectin; NSP, sodium carbonate-soluble pectin; HC, hemicellulose; CL, cellulose; SSA, specific surface area; APD, average pore diameter; WSP-DE, degree of esterification of WSP; CSP-DE, degree of esterification of CSP, WSP-Mw, molecular weight of WSP; CSP-Mw, molecular weight of CSP; NSP-Mw, molecular weight of NSP; WSP-R1, Ratio 1 of WSP; WSP-R2, Ratio 2 of WSP; WSP-R3, Ratio 3 of WSP; CSP-R1, Ratio 1 of CSP; CSP-R2, Ratio 2 of CSP; CSP-R3, Ratio 3 of CSP; NSP-R1, Ratio 1 of NSP; NSP-R2, Ratio 2 of NSP; NSP-R3, Ratio 3 of NSP; T_23_, transverse relaxation time of free water; A_23_, peak area corresponding to T_23_; DT, drying time."*" indicates a statistically significant difference (p<0.05).
